# Protein-species quantitative venomics: looking through a crystal ball

**DOI:** 10.1186/s40409-017-0116-9

**Published:** 2017-04-28

**Authors:** Juan J. Calvete, Daniel Petras, Francisco Calderón-Celis, Bruno Lomonte, Jorge Ruiz Encinar, Alfredo Sanz-Medel

**Affiliations:** 1 Structural and Functional Venomics Laboratory, Instituto de Biomedicina de Valencia, C.S.I.C, Jaime Roig 11, 46010 Valencia, Spain; 2 Skaggs School of Pharmacy & Pharmaceutical Sciences, University of California—San Diego, La Jolla, CA USA; 3 Department of Physical and Analytical Chemistry, University of Oviedo, Oviedo, Spain; 4 Instituto Clodomiro Picado, Facultad de Microbiología, Universidad de Costa Rica, San José, Costa Rica

**Keywords:** Snake venomics, Top-down proteomics, Top-down venomics, Protein species-resolved venomics, Absolute quantification, Inductively coupled plasma mass spectrometry

## Abstract

In this paper we discuss recent significant developments in the field of venom research, specifically the emergence of top-down proteomic applications that allow achieving compositional resolution at the level of the protein species present in the venom, and the absolute quantification of the venom proteins (the term “protein species” is used here to refer to all the different molecular forms in which a protein can be found. Please consult the special issue of *Jornal of Proteomics* “Towards deciphering proteomes via the proteoform, protein speciation, moonlighting and protein code concepts” published in 2016, vol. 134, pages 1-202). Challenges remain to be solved in order to achieve a compact and automated platform with which to routinely carry out comprehensive quantitative analysis of all toxins present in a venom. This short essay reflects the authors’ view of the immediate future in this direction for the proteomic analysis of venoms, particularly of snakes.

## Background

Rooted in a tradition of observation and description dating back at least to Aristotle, the study of natural phenomena (natural philosophy) involved for much of its history qualitative reasoning and explanations about nature. Aristotle’s conception of nature prevailed from the Middle Ages until the modern era. The precursor of modern science developed from natural philosophy with the introduction of the experimental method to make objective observations that can be verified by others as true or false. This approach was advocated by the Tuscan polymath Galileo Galilei (1564–1642) in 1638 with the publication of *Two New Sciences*. Galileo [1] revolutionized observational astronomy with his introduction and use of the telescope [2, 3]. Albert Einstein and Stephen Hawkins considered Galileo “the father of modern observational science”, as he based his science on careful observations, measurements, and controlled experiments. “The book of nature is written in the language of mathematics” is probably the most well-known genuine quote from Galileo Galilei. The scientific method exemplifies a mathematical understanding of nature that is the hallmark of modern natural scientists. Only by means of quantitative measurements can one arrive at the formulation of hypotheses and theories that account for the causal relationships or associations of the elements of a system.

Recent advances in high-throughput sequencing and mass spectrometry technologies have shifted the focus in biology from the measuring of a single protein, complex or pathway to the comprehensive analysis of all cellular components and their dynamic crosstalk. Beyond identification, it is important in most biological studies to know the quantity of a protein present in a sample. Although a single analytical method is usually insufficient to unravel in detail the complexity of living systems, perhaps the technical and conceptual framework that comes closest to this goal is mass spectrometry-based proteomics [4].

Established in the 1990s as a powerful though qualitative analytical technique [5–7], proteomics has undergone a revolution, and novel technologies for the systematic quantitative analysis of proteins have emerged coinciding with the turn of the century [8] and over the first decade of the XXI century [9]. These approaches have expanded our ability to acquire information from single proteins to proteomes, and promise that proteomes will soon be studied at a similar level of dynamic resolution as has been the norm for genome-wide gene expression using RNA microarrays and next-generation sequencing [10]. Label-free approaches have been applied to quantify snake venom proteomes [11, 12]. However, mass spectrometry is not inherently quantitative because of differences in the ionization efficiency and/or detectability of the many peptides in a given sample. This analytical limitation has sparked the development of methods to determine relative and absolute abundance of proteins in samples [9, 13].

## Peptide-centric mass spectrometry-based relative quantification

Mass spectrometry-based relative quantification techniques can be divided into two general categories: those that operate label-free, in which spectral counting or ion-intensity determinations of surrogate proteolytically-derived peptides represent a measure of the parent protein abundances [14], and those that use isotope-based methods for the comparative analysis of differential chemically or metabolically isotope-tagged proteomes [15]. Isotope-based methods incorporate heavy versions of specific molecules into the peptides, either by chemical derivatization or by metabolic labeling. Depending on the chemical derivatization technique employed, the differentially labelled peptides are quantified in MS or MS/MS mode [9, 16–24]. Thus, non-isobaric isotope-coded affinity tag (ICAT)-labeled peptides, metal-coded (MeCAT)-tagged peptides, residue-specific-tagged peptides such as ^13^C/^15^N dimethyl labeling of N-termini and ε-amino groups of lysine, and O^16^/O^18^ labelled peptides can be adequately quantified by MS.

On the other hand, peptides derivatized with isobaric tag for relative and absolute quantification (iTRAQ) or with isotopomer “tandem mass tags” (TMTs) require tandem MS-level quantification. These peptide-centric approaches are mainly used to quantify relative differences in peak intensity of the same analyte between multiple samples. Applications to venomics has been so far scarce, including the relative quantification of type A and type B venoms from the same species of *C. s. scutulatus* and the venoms from two geographically unrelated snakes from North and South America, *C. o. helleri* and *B. colombiensis*, respectively [25]. More recently, the comparative analyses of venom during the neonate-to-adult transition of *Bothrops jararaca* [26] and *Gloydius brevicaudus* were carried out [27].

The metabolic method stable isotope-labeling of amino acids in culture (SILAC) provides a powerful experimental strategy in certain circumstances (proteomic studies in cultured cell lines; in vivo quantitative proteomic using SILAC mice) [28]. However, it may not represent a feasible option when working with protein samples, such as venoms isolated from organisms that are not amenable to metabolic labeling.

## Isotope dilution mass spectrometry-based absolute quantification

Molecular mass spectrometry approaches using isotopic labeling have been extensively used over the last 15 years to quantify relative differences between a limited number of samples. However, transformation of the intensity signal ratios into absolute concentration values requires the use of species-specific internal calibration standards of controlled composition and certified concentration. Absolute proteomic quantification using isotopic peptides entails spiking known concentrations of synthetic, heavy isotopologues (e.g. AQUA—**a**bsolute **qua**ntification-peptides; QconCAT—**q**uantification **concat**amer) of the proteotypic target peptides into an experimental sample, before the digestion step, to determine the intensity ratio (isotope dilution) of spiked and target peptides by LC-MS or LC-MS/MS [29–33]. The abundance of the target peptide in the experimental sample is back calculated to the initial concentration of the standard using a pre-determined standard curve to yield the absolute quantification of the target peptide.

**Fig. 1:**
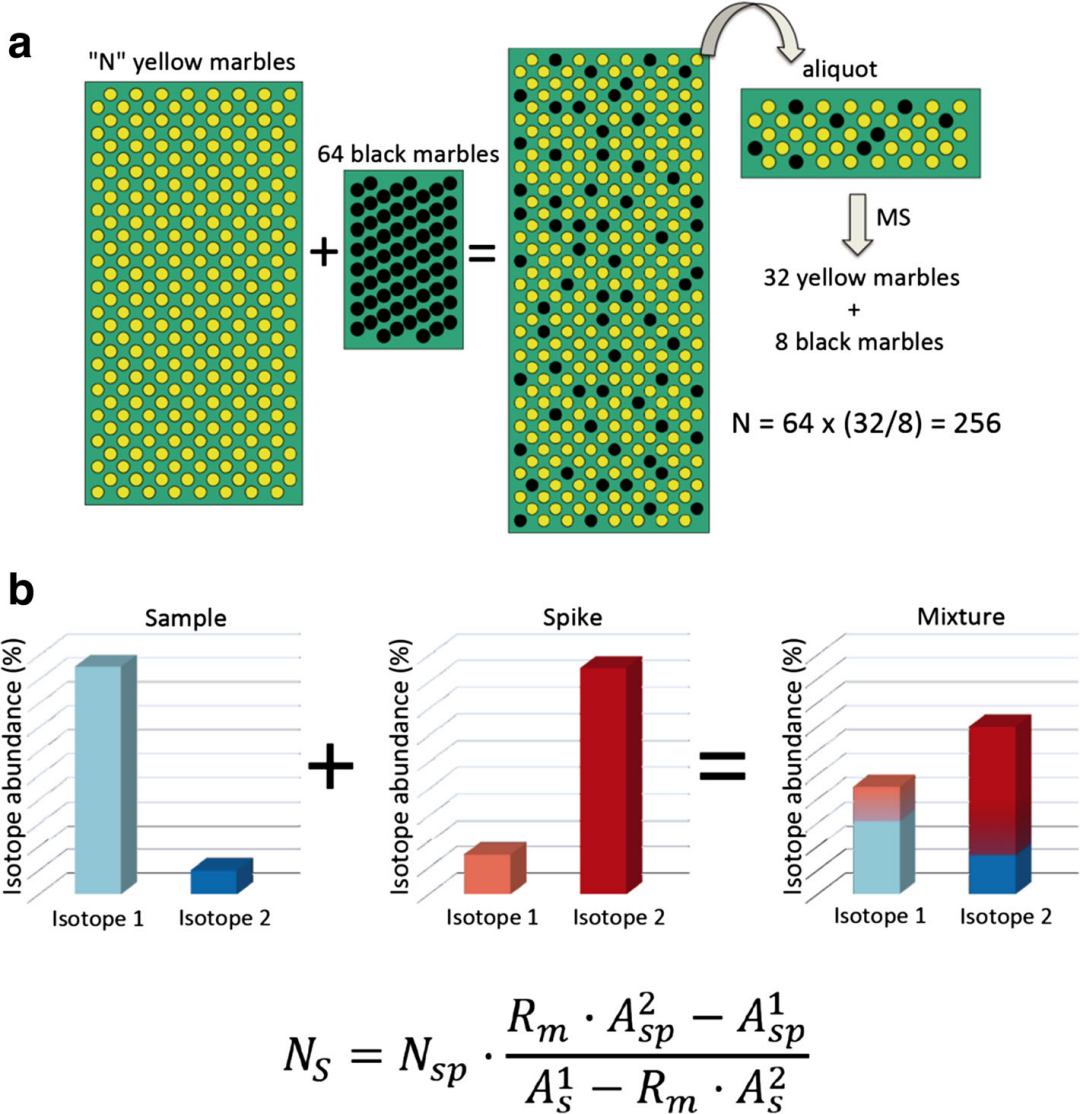
The principle of isotope dilution. **a** Simplified cartoon (adapted from Alonso and González [33]) illustrating the principle of absolute quantification by dilution. The addition of a known amount of an internal standard (*black marbles*) to a sample containing an unknown (N) number of an analyte (*yellow marbles*) changes the concentration of the analyte. By determining the ratio of internal standard to unknown analyte in the resulting mixture, it is possible to back-calculate the amount of the analyte present in the sample. **b** A more complex situation arises in isotope dilution analysis when the sample, of natural isotopic composition, is mixed with an isotopically enriched spike. The image illustrates an example for an element containing two different isotopes (1 and 2). The resulting isotopic composition of the mixture to be measured is the combination of the sample’s and spike’s individual isotopic compositions and their molar ratios because the moles of the element in the mixture is the sum of the moles coming from the sample and the spike. If the number of moles added with the spike (N_sp_), as well as the isotopic composition of sample and spike (abundances of the isotopes 1 and 2 in the sample and spike: *A*_*s*_^1^, *A*_*s*_^2^ and *A*_*sp*_^1^, *A*_*sp*_^2^, respectively) are known, it is hence possible to determine the number of moles of the element in the sample (N_s_) from the measurement of a single isotope ratio in the mixture (R_m_)

Analytical application of the radiotracer method represents the forerunner of isotope dilution. This method was developed in the early 20^th^ century by the Hungarian chemist George de Hevesy [34], for which he was awarded the Nobel Prize in Chemistry in 1943. Isotope dilution mass spectrometry is a direct ratio method that has been identified by the Consultative Committee for Amount of Substance (CCQM) of the International Committee for Weights and Measures (CIPM) to have the potential to be a primary method. Scanning modes available in tandem mass analyzers, such as selected reaction monitoring (SRM) and parallel reaction monitoring (PRM), can be applied to targeted proteomic workflows in combination with isotopically-labeled versions of proteotypic peptides, which uniquely represent target proteins or a protein isoform, to monitor a selection of proteins of interest with high sensitivity, reproducibility and quantitative accuracy [35–39]. However, these methods are very laborious and costly, as they require the synthesis and characterization of at least one individual isotopic standard for each target protein, making targeted proteomic approaches impractical, particularly in venom analysis. A possible alternative to overcome these limitations is a well-known technique in the field of bioinorganic analysis: inductive coupled plasma mass spectrometry (ICP-MS) combined with stable-isotope dilution. Figure 1 illustrates the principle of isotope dilution for absolute quantification.

## ICP-MS

**Fig. 2:**
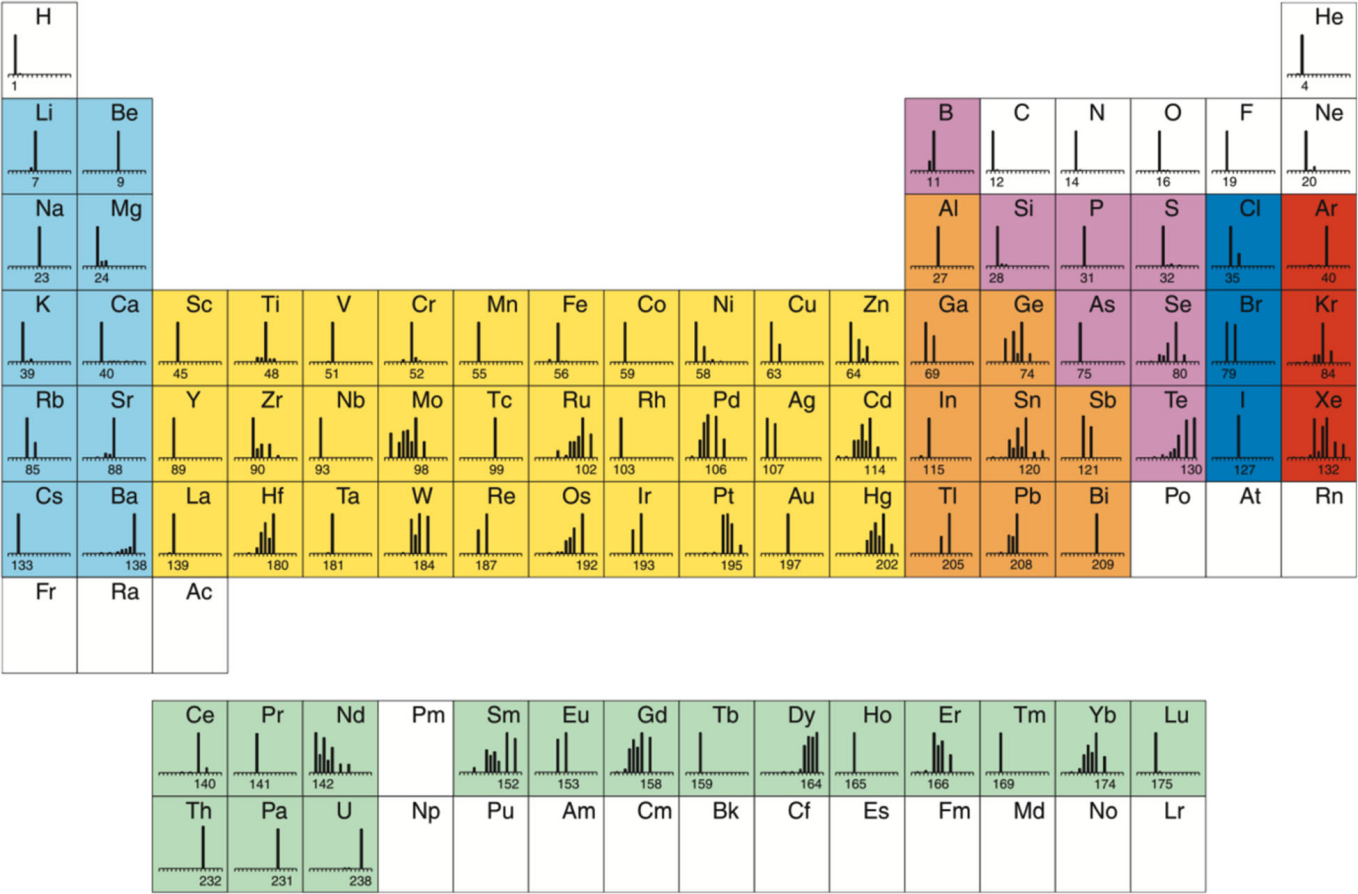
Color-coded groups of elements traditionally determined by ICP-MS (courtesy of PerkinElmer, Inc.). *Light blue*, alkali earth and alkaline earth; *yellow*, transition metals; *orange*, other metals; *magenta*, metalloids; *dark blue*, halogens; *red*, noble gases; *pale green*, rare earth elements of the Lanthanide and Actinide series

ICP-MS is a type of elemental mass spectrometry introduced by Houk et al. [40] in 1980. Commercially introduced soon after 1983 for elemental determinations, ICP-MS has become the most powerful analytical tool for tracing elemental analysis, enabling robust determinations of metals, semimetals and several nonmetals (and their different isotopes) at concentration levels as low as one part in 10^15^ (part per quadrillion, ppq) using adequate non-interfered low-background isotopes [41] (Fig. 2). This is achieved by atomizing and ionizing the sample in a “hard” ion source, inductively coupled argon plasma. Once the elemental ions from the sample enter the mass spectrometer, they are separated by their mass-to-charge ratio. The most commonly used type of mass spectrometer is the quadrupole (Q) mass filter.

The potential of ICP-MS for screening simultaneously multiple metals, semimetals and biologically important nonmetals (e.g., S, P, I), naturally occurring in proteins, and its capability of achieving absolute protein quantifications via determinations of heteroatoms have been reviewed [42, 43]. Among these elements, sulfur results of particular relevance in proteomics (and specifically in venomics). Incorporated into the amino acids methionine and cysteine, the element sulfur is present in almost all toxin classes, particularly in small proteins whose global folds are stabilized primarily by the formation of disulfide bonds [44]. Mass spectrometric determination of cysteine (in SH and S–S forms) content represents a useful proxy for the preliminary classification of toxins into protein families [45].

The omnipresence of sulfur in venom proteins, and the fact that they can be efficiently separated by reversed-phase high-performance liquid chromatography (RP-HPLC), makes the absolute protein quantification using sulfur analysis by ICP-MS feasible. The main advantage of this approach is that only one generic sulfur-containing standard (i.e., one isotopically labeled sulfur spike such as ^34^S-sulphate) is required to quantify each and all proteins of a venom proteome provided that they are completely separated and their amino acid sequences are known [46]. Moreover, the recent introduction of the tandem ICP-MS concept (triple quadrupole QQQ mass analyzer) enabled limits of detection (LODs) in the low femtomole range for S-containing peptides/proteins [47]. Of course, peak purity is here a pre-requisite as ICP-MS-based elemental detection cannot distinguish if sulfur comes from one or another protein or another compound present in the sample.

Moreover, the amino acid sequence information and sulfur/protein stoichiometry are needed to transform the total ICP-MS measured peak sulfur mass content into intact protein concentration (e.g., as moles of toxin per gram of venom). This way of expressing the data has more biological sense than “g of toxin/g of total venom proteins” derived by monitoring the RP-HPLC eluate with UV-VIS at 215 nm, since the number of toxic molecules, rather than their mass, is responsible for the biological effects of the venom.

**Fig. 3:**
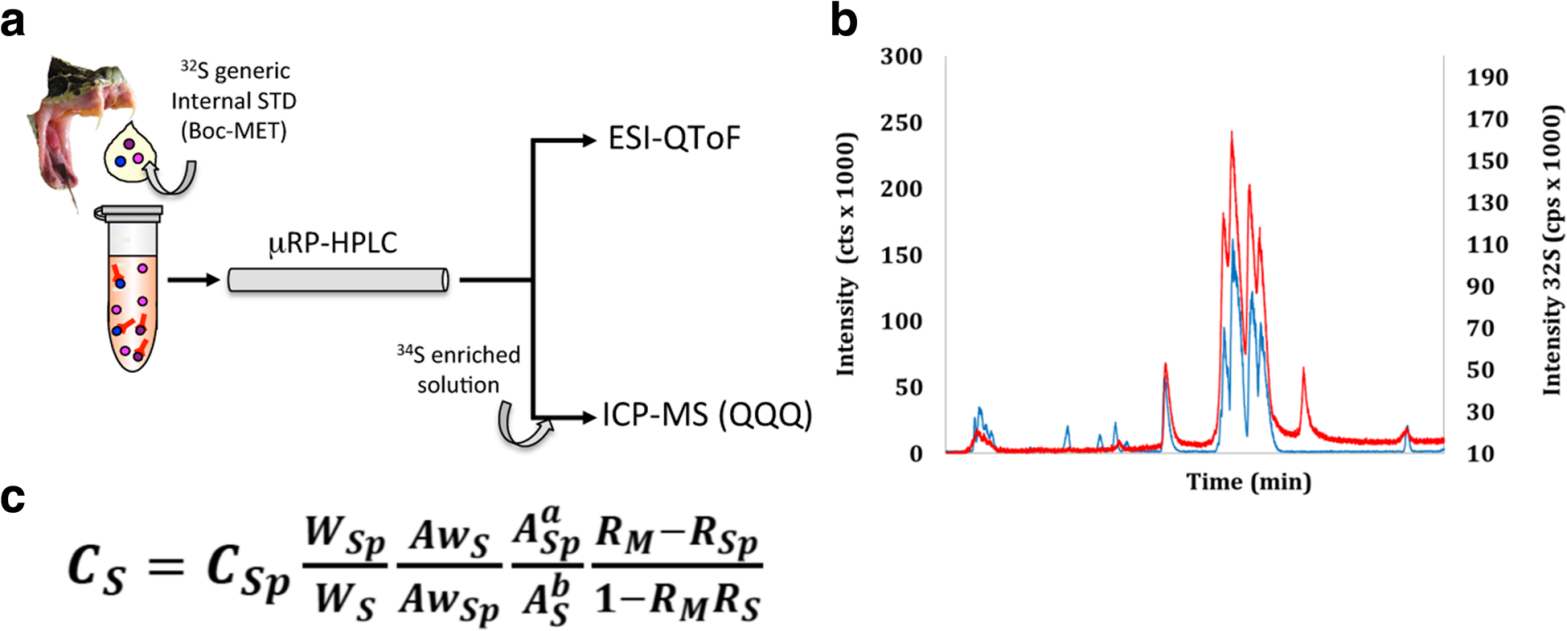
**a** Scheme of the parallel hybrid RP-μHPLC-ICP-QQQ with on-line ^34^S isotope dilution and LC-ESI-QToF analyses for the absolute quantitative analysis of the major toxins identified by mass profiling in the venom of the Mozambique spitting cobra, *Naja mossambica* [48]. **b** Overlay of ESI-QToF protein (*blue trace*, *left* *y-axis*) and ICP-QQQ ^32^S (*red trace*, *right* *y-axis*) chromatograms allowed peak correlation of ICP-QQQ and ESI-QToF spectra. The resolution (50000) and mass accuracy (0.2 ppm) of the ESI-QToF instrument employed allowed accurate protein identification by mass profiling, and the observed excellent peak patterns matching enabled correlating molecular peak identity and elemental S quantitation. Relation of the integrated mass flow peak areas results in sulfur quantification using the equation displayed in (**c**) panel. C_S,_ sulfur concentration in the sample; C_Sp_, sulfur concentration in the ^34^S spike; W_S_ and W_Sp_, weighted mass of sample and spike, respectively; Aw_S_, sulfur atomic weight in the sample; Aw_Sp_, sulfur atomic weight in the spike; A^a^_Sp_, ^34^S abundance in the spike; A^b^_S_, ^32^S abundance in the sample, R_M_, the ^32^S/^34^S ratio in the mixture; R_Sp_, the ^32^S/^34^S ratio in the spike; and R_S_, the ^32^S/^34^S ratio in the sample

Very recently, Calderón-Celis et al. [48] have reported the application of RP-μHPLC-ICP-QQQ and on-line ^34^S isotope dilution analysis for the absolute quantitative analysis of the major toxins comprising the venom proteome of the Mozambique spitting cobra, *Naja mossambica*. Identification of the toxins eluting along the chromatographic separation was carried out by ESI-MS mass profiling in parallel to the ICP-MS measurements, matching the recorded isotope-averaged molecular masses to the calculated masses for mature *Naja* spp. proteins deposited in the non-redundant NCBI database and to *N. mossambica* venom proteins previously identified by peptide-centric venomic analysis [49]. The results indicated that elemental MS, via tandem ICP-MS (QQQ) represents a direct and accurate methodology for absolute quantification of venom proteomes. A schematic of this hybrid (molecular and elemental) workflow is displayed in Fig. 3.

A note of caution: this approach works well for proteins without unpredictable PTMs, as is the case of the major toxins of many species of elapids (such as 3FTxs, PLA_2_s, Kunitz-fold proteins, cysteine-rich secretory proteins, C-type lectin-like proteins), but may be impracticable for other proteins, eg. toxins bearing complex PTMs as glycosylation (i.e. snake venom metalloproteinases, snake venom serine proteinases). Identification of these proteins should be based on internal sequence determination, usually performed using bottom-up MS/MS approaches.

The trend towards hybrid configurations of mass analyzers has dominated recent advances in instrumentation. Hybrid mass spectrometry systems use various designs of *in-space* beam-type and *in-time* ion-trapping spectrometers to combine the different performance characteristics offered by the individual mass analyzers into one instrument. The incorporation of ICP-MS into current and novel mass spectrometry workflows may open the door to a synergistic pair’s work. That is, a judicious combination of elemental and molecular MS approaches could provide enhanced robustness, sensitivity, analytical speed and overall performance through the parallel identification and absolute quantification of heteroatom-bearing peptides and proteins.

## Top-down venomics

Bottom-up venomics platforms (outlined in the study by Lomonte et al. [50]) usually provide incomplete protein sequence coverage, not allowing distinguishing between different protein species, particularly proteoforms or closely related isoforms of toxin family members [51–53]. In addition, proteolytic digestion eliminates the connectivity between intact proteins and the tryptic peptides they yield, complicating computational analysis and biological interpretations. To a certain extent, locus-specific assignments can be achieved by using an homologous snake venom gland transcriptome as a database for the assignment of mass spectra [54, 55].

Top-down mass spectrometry has the potential to eliminate the shortcomings of bottom-up workflows [56, 57]. Top-down MS is typically performed on Fourier-transform ion trap mass spectrometers, which offer the ultra-high mass resolution needed to achieve isotope resolution for charged state determinations of fragment ions in MS/MS experiments. Our typical top-down venomics workflow involves: front-end fractionation of complex disulfide-bond-reduced protein mixtures; electrospray ionization of the intact polypeptides to generate charged particle that can be manipulated and dissociated inside the mass spectrometer; high-resolution mass spectral data acquisition at precursor and fragment levels; and bioinformatic data processing by spectra searching/scoring against a species-specific database using various software tools to match the product ion dataset with the primary sequences of the proteins, including all modifications that affect their masses [58, 59].

After more than 20 years of mass spectrometry-based bottom-up proteomics, top-down proteome analysis is gaining momentum [60]. However, there are still limitations on front-end fractionation of complex mixtures and instrumentation-related challenges behind its implementation, particularly on high mass proteins [60]. Top-down venomics is in its infancy. Only very recently reports on *Ophiophagus hannah* [61, 62] and *Dendroaspis* (*angusticeps* and *polylepis*) [63] venoms have proved that top-down venomics represents a fast and accurate tool for locus-specific assignment of many previously undetected protein species (iso- and proteoforms) of many known venom proteins, including the identification and precise location of acetylated lysine residues [63].

**Fig. 4:**
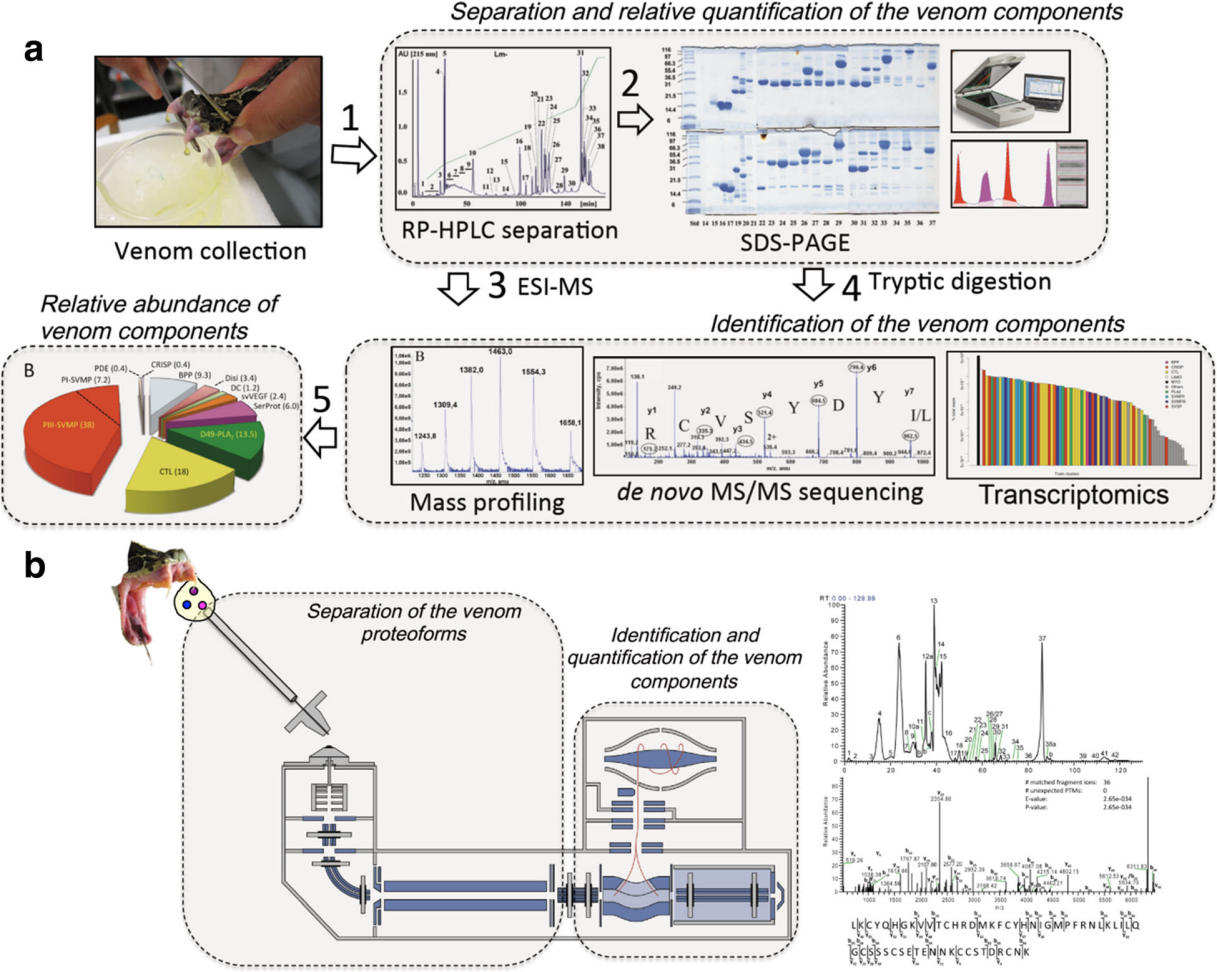
Scheme of (**a**) *bottom-up* and (**b**) *top-down* *venomics* workflows used in the authors’ laboratories. In *bottom-up* *venomics*, offline pre-MS venom fractionation is used to quantify the relative abundance of the venom components, whereas in the *top-down* approach proteoform identification and quantification is performed inside a high-resolution ion trapping mass spectrometer. A practical consequence of the *top-down* *configuration* is the possibility of automating the whole process, reducing the analysis time from weeks (*bottom-up venomics*) to hours

In comparison to bottom-up approaches, where off-line pre-MS decomplexation of the venom proteome by RP-HPLC/SDS-PAGE represents the Rosetta Stone to quantify the venom components [55, 64] (Fig. 4a), top-down venomics offers the potential of simultaneously identifying and quantifying the whole venom proteome at protein species resolution using labeling strategies or label-free methods (Fig. 4b) [56, 65–68].

## A quick look through the glass

Research on venoms has been continuously enhanced by advances in technology. The increased use of sensitive proteomics techniques over the last decade has revolutionized venomics research [69]. Achieving complete pre-ICP-MS separation and structural characterization of all the components of complex proteomes, such as snake venoms, represents an important challenge of contemporary venom analysis. Integrated with comprehensive venom gland transcriptomic and/or genomic datasets, novel computational tools for optimizing protein identification results, and with advances in MS instrumentation, dissociation strategies and bioinformatic tools, it is not unreasonable to speculate that top-down venomics approaches represent the cornerstone for achieving the challenging task of full description of venom proteomes [70–74].

Establishing the link between genotype and phenotype requires understanding the molecular basis of complex adaptive traits, such as venoms, which in turn demands both qualitative and quantitative comparisons of the temporal and spatial patterns of venom variation. The study of the geographic distribution of genetic variation within a species provides the basis for formulating hypotheses to explain the ecological processes responsible for the evolution of biodiversity, and to define the boundaries of species. Besides proving a molecular perspective for evolutionary studies on venoms, protein species-resolved absolute quantitative approaches will also have a great impact in other venomics disciplines such as toxicovenomics, ecological venomics, and antivenomics [75–83].

## Conclusions

The application of next generation sequencing and high resolution mass spectrometry to study animal venoms has grown steadily in recent years, and quantitative locus-resolved venom proteomes will increasingly be the goal of next-future venomics. In particular, the integration of top-down venomics, toxicovenomics, absolute quantitation, venom gland RNAseq and comparative snake genomics into a comprehensive evolutionary framework will revolutionize the field of molecular toxinology in the coming years. Understanding the natural history and evolutionary pressures that shaped the complexity of extant snake venoms is of applied importance for unveiling the molecular mechanisms that underlie venom variability, exploring the enormous potential of venoms as sources of chemical and pharmacological novelty, but also for the manufacture of novel, safer and more effective therapeutic antivenoms of broader therapeutic use [81, 83–85]. Clearly, implementing top-down and absolute quantification approaches into next-generation venomic workflows promises a quantitative leap in the study of venoms and a bright future to the field of integrative venomics [86].

## Abbreviations

CCQM: Consultative Committee for Amount of Substance
CIPM: International Committee for Weights and Measures
ICAT: Isotope-coded affinity tag
ICP-MS: Inductively coupled plasma mass spectrometry
iTRAQ: Isobaric tag for relative and absolute quantification
LODs: Limits of detection
MeCAT: Metal-coded affinity tag
PRM: Parallel reaction monitoring
RP-HPLC: Reversed-phase high-performance liquid chromatography
SRM: Selected reaction monitoring
TMTs: Tandem mass tags
